# Large Language Models and the Wisdom of Small Crowds

**DOI:** 10.1162/opmi_a_00144

**Published:** 2024-05-20

**Authors:** Sean Trott

**Affiliations:** Department of Cognitive Science, University of California, San Diego, San Diego, CA, USA

**Keywords:** dataset, psycholinguistic resource, wisdom of the crowd, large language models, ChatGPT

## Abstract

Recent advances in Large Language Models (LLMs) have raised the question of replacing human subjects with LLM-generated data. While some believe that LLMs capture the “wisdom of the crowd”—due to their vast training data—empirical evidence for this hypothesis remains scarce. We present a novel methodological framework to test this: the “number needed to beat” (NNB), which measures how many humans are needed for a sample’s quality to rival the quality achieved by GPT-4, a state-of-the-art LLM. In a series of pre-registered experiments, we collect novel human data and demonstrate the utility of this method for four psycholinguistic datasets for English. We find that NNB > 1 for each dataset, but also that NNB varies across tasks (and in some cases is quite small, e.g., 2). We also introduce two “centaur” methods for combining LLM and human data, which outperform both stand-alone LLMs and human samples. Finally, we analyze the trade-offs in data cost and quality for each approach. While clear limitations remain, we suggest that this framework could guide decision-making about whether and how to integrate LLM-generated data into the research pipeline.

## INTRODUCTION

The advent of powerful Large Language Models (LLMs) like ChatGPT has raised the question of whether these new tools can accelerate scientific progress (Grossmann et al., 2023; Korinek, 2023; Sourati & Evans, 2023). In theory, LLMs could help researchers brainstorm ideas, generate hypotheses, conduct literature reviews, analyze data, and perhaps—in the case of human behavioral research—even serve as “research subjects” (Dillion et al., 2023; Grossmann et al., 2023). Collecting data from human participants is a major bottleneck in terms of both time and money; online data collection tools have ameliorated this somewhat, but recent evidence has raised concerns about the quality of data collected on some platforms (Veselovsky et al., 2023). The idea of using LLMs as *in silica* “model organisms” has thus generated intense interest (Aher et al., 2023; Dillion et al., 2023; Gilardi et al., 2023; Hagendorff et al., 2023; Jain et al., 2024)^1^; some researchers have even argued that LLMs are actually better “participants” than human workers (Gilardi et al., 2023), perhaps because they capture the “wisdom of the crowd” (Dillion et al., 2023). Despite considerable debate (Crockett & Messeri, 2023; Harding et al., 2023), however, there remains a dearth of empirical evidence directly comparing the viability of LLMs to the *de facto* alternative: a sample of human participants. More precisely: do LLMs actually capture the wisdom of the crowd (Dillion et al., 2023)—and if so, what is the *size* of that crowd?

Human behavioral research almost always relies on a sample of human participants. Although no sample is perfectly representative of the underlying population of interest, researchers have long operated on the premise that a random sample of sufficient size can reduce sampling error such that relevant theoretical inferences can be made (e.g., rejecting a null hypothesis or estimating a regression coefficient)^2^. As sample size increases, the distribution of hypothetical *sample means* of a given size displays lower variance (and becomes increasingly normal), thereby decreasing the margin of error in estimates calculated from that sample. A larger sample is thus said to have larger power than a small sample, i.e., a better chance of correctly detecting a true effect.

This relates to the so-called “wisdom of the crowd” (Stroop, 1932), a phenomenon whereby the average of many guesses (e.g., of the number of jelly beans in a jar) is often better than the majority of those guesses on their own. Thus, when psychology researchers collect ratings or “norms” for their stimuli, they typically collect more than one rating for each linguistic stimulus, then calculate the *average* of those ratings (Brysbaert et al., 2014; Lynott et al., 2020; Winter et al., 2024); this insight is also the foundation of ensemble models in machine learning (Dietterich, 2000). In both cases, the assumption is that increasing the set of decision-makers per item (e.g., a stimulus) will generally increase the quality of the resulting average decision, provided the sample is sufficiently diverse—though importantly, the increase in quality is not necessarily linear (i.e., there are “diminishing returns”).

These background assumptions are crucial to the approach taken in the current work. Recent evidence (Dillion et al., 2023; Trott, 2024; Trott et al., 2023) suggests that the behavior of LLMs correlates surprisingly well with the aggregate behavior of human subjects in published psychology research—in some cases, LLM-generated behavior is *more correlated* with the human average than the average human subject is (Trott, 2024). As Dillion et al. (2023) point out, this could be a result of how LLMs are trained: “The ‘minds’ of language models are trained on vast amounts of human expression, so their expressions can indirectly capture millions of human minds” (pg. 3).

More precisely, LLMs like GPT-3 are trained on hundreds of billions of word tokens (Brown et al., 2020), which is many orders of magnitude more than the average human encounters during development (Hart & Risley, 1992; Hosseini et al., 2024). During training, LLMs learn to predict upcoming tokens (e.g., “water”) from a previous string of tokens (e.g., “I need a glass of ___”). The behavior that LLMs learn to produce (i.e., predicted tokens) is in some sense the aggregate of their input; their input, in turn, is in certain ways much larger than that of an individual human’s.^3^ If this line of reasoning is correct (Dillion et al., 2023), it is possible that for select domains, LLM-generated behavior is not only correlated with human behavior, but it may even approximate a *sample mean* that reflects a better estimate of the underlying *population parameter* than individual human judgments.

Determining whether or not this is true—and under which conditions—is of central importance to human behavioral researchers. If it is true, then researchers could save considerable time and money by augmenting their human sample with LLMs (Dillion et al., 2023). Further, researchers would benefit from knowing the approximate “sample size” that an LLM’s “sample mean” reflects, i.e., the “number needed to beat” (NNB) an LLM’s data quality for a given task. Whereas if it is not true, then current LLMs may not be effective in addressing one of the major bottlenecks in behavioral research (data collection).

In three pre-registered experiments, we compared the quality of judgments elicited from an LLM to the quality of judgments from novel human samples of varying size (i.e., a single human, two humans, etc.). We focused on four psycholinguistic datasets: judgments about the concreteness of different English words in context, taken from the Glasgow Norms (Scott et al., 2019); judgments about the valence of different English words in context, also taken from the Glasgow Norms (Scott et al., 2019); judgments about the relatedness of ambiguous English words in context, i.e., RAW-C (Trott & Bergen, 2021); and judgments about the iconicity of various English words (Winter et al., 2024). These datasets were selected because prior work (Trott, 2024) suggested that judgments from GPT-4, a state-of-the-art LLM, were more correlated with the gold standard (i.e., previously published ratings) than were individual human ratings. We then collected novel human data for each dataset, and asked: how many human judgments must be included in a sample, such that the resulting sample mean achieves the same degree of correlation with the gold standard as does GPT-4’s ratings (i.e., the “number needed to beat”, or NNB)? Further, we experimented with two augmentation or “centaur” methods, in which human ratings were combined with LLM ratings. Together, these results allowed us to determine the size of the crowd that an LLM’s judgments reflects for a given task.

Pre-registered analyses can be found on OSF for the Glasgow concreteness norms (https://osf.io/cdt6r), the Glasgow valence norms (https://osf.io/y3z4p/), the RAW-C dataset (https://osf.io/cdt6r), and the iconicity dataset (https://osf.io/dvysw). Additionally, code to reproduce the analyses and figures in the text can be found on GitHub (https://github.com/seantrott/llm_clt/).

## METHODS

### Datasets

As “gold standard” datasets, we used RAW-C (Trott & Bergen, 2021), a portion of the Glasgow Norms (Scott et al., 2019), and a subset of a recently published dataset of iconicity judgments (Winter et al., 2024). The original RAW-C dataset contains relatedness judgments about ambiguous English words in different contexts. Specifically, there are 112 English words, each of which appears in four sentential contexts (two sentences for each of the primary meanings of that ambiguous word); each word has six possible comparisons, for a total of 672 sentence pairs (e.g., “She liked the marinated *lamb*” vs. “She liked the friendly *lamb*”). Participants rated the relatedness of the target ambiguous word (e.g., “lamb”) on a scale from 1 (totally unrelated) to 5 (same meaning). In the original dataset, the average number of judgments per sentence was 12.8 (median = 13), and ranged from 4 to 23.

For the Glasgow Norms, we focused on ratings of concreteness and valence, specifically for *contextualized* judgments about ambiguous words (e.g., “bow (ship)” or “bow (arrow)”). There were 379 ambiguous words appearing in different contexts, for a total 871 ratings overall. For concreteness, participants rated each word meaning according to its concreteness on a scale from 1 (very abstract) to 7 (very concrete). According to the original dataset, the average number of judgments per word was 33.4 (median = 34), and ranged from 22 to 36. For valence, participants rated each word according to how positive or negative it was, ranging from 1 (very negative) to 7 (very positive). According to the original dataset, the average number of judgments per word was 33.6 (median = 34), and ranged from 24 to 36.

The iconicity dataset (Winter et al., 2024) contains iconicity judgments for 14,776 English words. Because it was published in 2023, it is less likely to have been part of GPT-4’s training data than the other datasets (though tests of data contamination did not reveal evidence consistent with contamination for other datasets either; see Trott (2024) for more details). Participants rated the iconicity of words on a scale from 1 (not iconic at all) to 7 (very iconic). According to the original dataset, after applying exclusion criteria, each word was rated by at least ten participants; the average number of ratings per word was 10.9 (median = 10). To make replication tractable, we used a subset of the original 14,776 norms, randomly sampling 800 words from the original list.

Each original dataset is publicly available online: RAW-C (https://github.com/seantrott/raw-c), the Glasgow Norms (https://link.springer.com/article/10.3758/s13428-018-1099-3; see Appendix), and the iconicity norms (https://osf.io/qvw6u/).

### GPT-4 Rating Elicitation

Previous work (Trott, 2024) used the Python OpenAI API to access the GPT-4 model and elicit ratings for a range of psycholinguistic datasets, including the four analyzed here. In each case, GPT-4 was prompted using instructions matched to those given to human participants in the original study as closely as possible, with a temperature of 0. A temperature of 0 should theoretically result in deterministic or near-deterministic^4^ responses, i.e., a form of maximum likelihood estimation. (The question of capturing variance in responses, as opposed to the highest-probability token, is explored in the General Discussion.) Note that the version of GPT-4 used for all experiments was *gpt-4-0613*, which according to the OpenAI documentation (https://platform.openai.com/docs/models/gpt-4-and-gpt-4-turbo) has a training data cutoff of September, 2021.

GPT-4 was allowed to generate up to 10 tokens in response; responses were processed to extract numerical ratings (e.g., “1” would be extracted from the response “1 (totally unrelated)”). Each stimulus item was presented to GPT-4 in isolation to avoid contamination between responses. Along with the stimulus itself, the entire set of instructions was presented in the prompt.

### Human Experiments

Our main goal was to determine how many human participants were needed for their average rating to match GPT-4’s correlation with the gold standard dataset. Here, “gold standard” was taken to be the original published datasets (Scott et al., 2019; Trott & Bergen, 2021; Winter et al., 2024).

#### List Creation.

There were too many items for each participant to provide ratings for the entire dataset. However, in order to calculate Number Needed to Beat (NNB), it was necessary to ensure that the exact same subset of items was rated by more than one participant, i.e., so that samples of various sizes could be compared against GPT-4’s correlation for that specific subset of items. Thus, we split each dataset into a series of lists. RAW-C was split into 8 lists of 84 items each, the Glasgow Norms were split into 17 lists of approximately 50 items each (for both valence and concreteness), and the 800 randomly-sampled items from the iconicity dataset were split into 10 lists of 80 items each. Items were randomly assigned to each list.

#### Participants.

Participants were recruited through the Prolific recruiting platform (Palan & Schitter, 2018). As described in both pre-registrations, our target number of participants was approximately 10 per stimulus list, i.e., 170 participants for each of the Glasgow Norms (concreteness and valence), 80 participants for RAW-C, and 100 participants for the iconicity norms.

We deliberately over-sampled in each case, as we anticipated needing to exclude data. We recruited a total of 200 participants for each of the Glasgow Norms (concreteness and valence), 102 participants for RAW-C^5^, and 200 participants for the iconicity norms. In order to qualify for the study, participants had to reside in the United States and be native speakers of English. In the Glasgow concreteness replication, there were 104 male participants (92 female and 4 unknown), with an average age of 34.66 (*SD* = 10.94, median = 32.5). In the RAW-C replication, there were 55 males (46 females, and 1 “prefer not to say”), with an average age of 36.35 (*SD* = 13, median = 32). For the Glasgow valence replication, there were 96 male participants (102 female and 2 unknown), with an average age of 37.8 (*SD* = 12.78, median = 35). For the iconicity replication, there were 102 female participants (92 male, 5 non-binary, and 1 prefer not to say), with an average age of 38.5 (*SD* = 13.28, median = 34.5).

After applying our pre-registered exclusion criteria, there were 181 participants in the Glasgow Norms concreteness replication, 142 participants in the Glasgow Norms valence replication, 94 participants in the RAW-C replication, and 168 participants in the iconicity replication.

Participants were paid $2 to participate in the iconicity study and $3 to participate in the other studies. The median completion time was 5:50 minutes for the Glasgow concreteness replication (a rate of $30.86 per hour), 5:29 minutes for the Glasgow valence replication (a rate of $21.88 per hour), 10:22 minutes for the RAW-C replication (a rate of $15.72 per hour), and 8:28 minutes for the iconicity task (a rate of $14.17 per hour).

#### Procedure.

After indicating consent, participants read a series of instructions about their task. These instructions were designed to mirror the original instructions for the Glasgow Norms, the RAW-C task, and the iconicity dataset. For the Glasgow Norms, participants were told they would be rating the concreteness (or valence) of approximately 50 words; they were given an example of a concrete concept and an abstract concept (or a positive or negative concept). They were also told that words mean different things in different contexts. There were similar instructions for the RAW-C task, only with an example about word relatedness instead of word concreteness. The examples that appeared in the instructions did not appear in any of the actual stimulus lists. Finally, the iconicity task followed the instructions of Winter et al. (2024), providing a definition of iconicity, as well as examples of words that were highly iconic and words that were not iconic. Both the examples and the instructions were copied from Winter et al. (2024).

Then, each participant was randomly assigned to one of the stimulus lists. In this main part of the task, participants rated a series of words (or sentence pairs) according to their concreteness (or valence or relatedness). Each stimulus appeared on a separate page, and the participant advanced to the next stimulus after rating the current stimulus and pressing a “Next” button.

**Figure A1.**
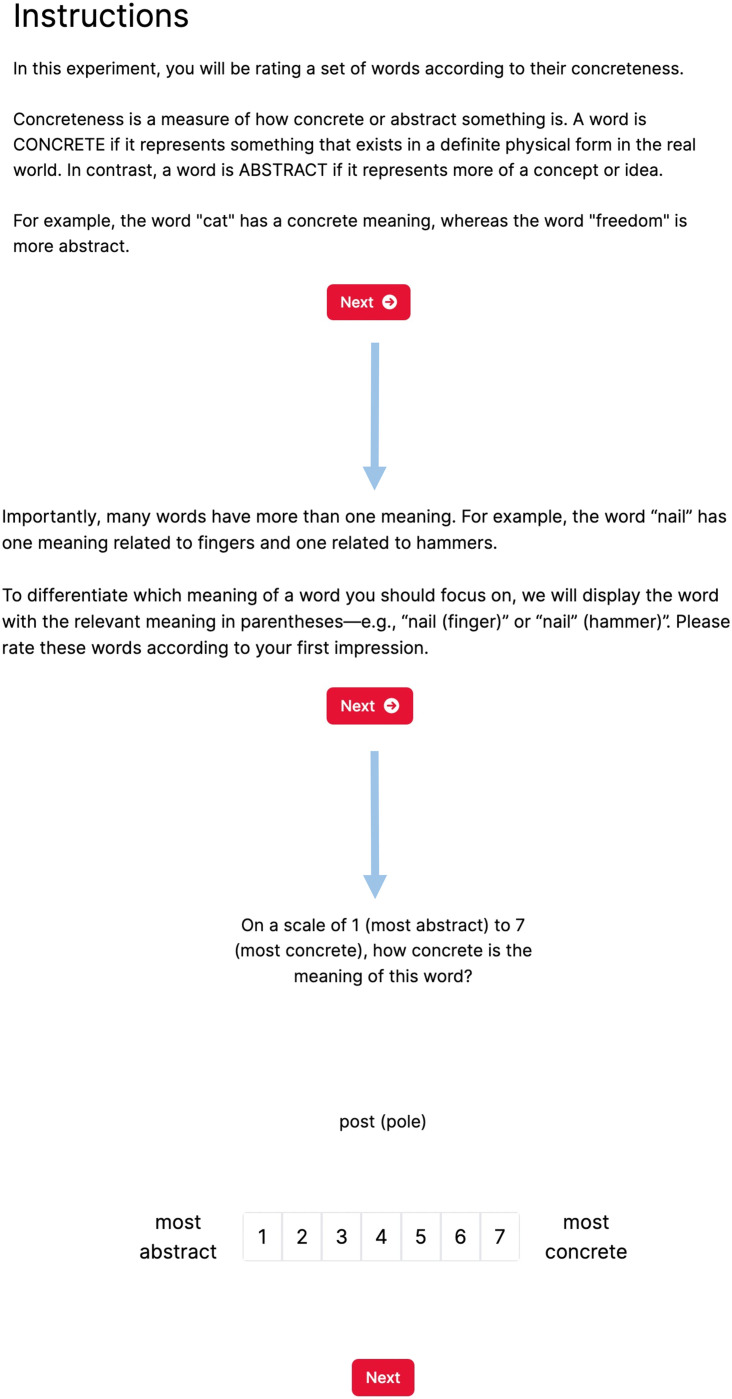
Graphical visualization of the instructions and a trial screen for the Glasgow concreteness replication.

Each experiment, except for the iconicity experiment, also contained a “catch trial”. In the concreteness and valence replications, the stimulus was replaced by a sentence instructing the participant to answer “1”; in the RAW-C replication, participants had to rate the relatedness of the word “rose” in two identical sentences (i.e., such that the correct answer was always “5”). See Figure A1 for a visual illustration of the primary task.

Finally, after completing the main task, participants read a debrief screen explaining the purpose of the experiment. All participants were paid for their participation regardless of whether they answered the catch trials correctly.

All replication studies were designed using the Gorilla platform (Anwyl-Irvine et al., 2020).

#### Exclusion Procedure.

As described in our pre-registrations, participants were excluded from the primary analysis if they answered the catch trial incorrectly (except for the iconicity dataset, which did not include a catch trial).

They were also excluded using a leave-one-out correlation procedure. In this procedure, we calculated the correlation between each participant’s set of ratings and the ratings in the originally published dataset. Following Winter et al. (2024), we excluded participants whose correlations with the gold standard were less than 0.1.

We also applied two other exclusion criteria to the iconicity dataset specifically to replicate the procedure applied in Winter et al. (2024). Specifically, we excluded trials with a response time under 500 ms, as well as “straight-liners” (i.e., participants who responded the same way to over 80% of their trials).

All exclusion criteria were designed to ensure data quality, i.e., to make sure that GPT-4’s ratings did not enjoy an unfair “advantage” due to inclusion of poor quality human data.

#### Declarations.

All studies were conducted with the approval of the organization’s Institutional Review Board.

### Analysis

**Figure 1.**
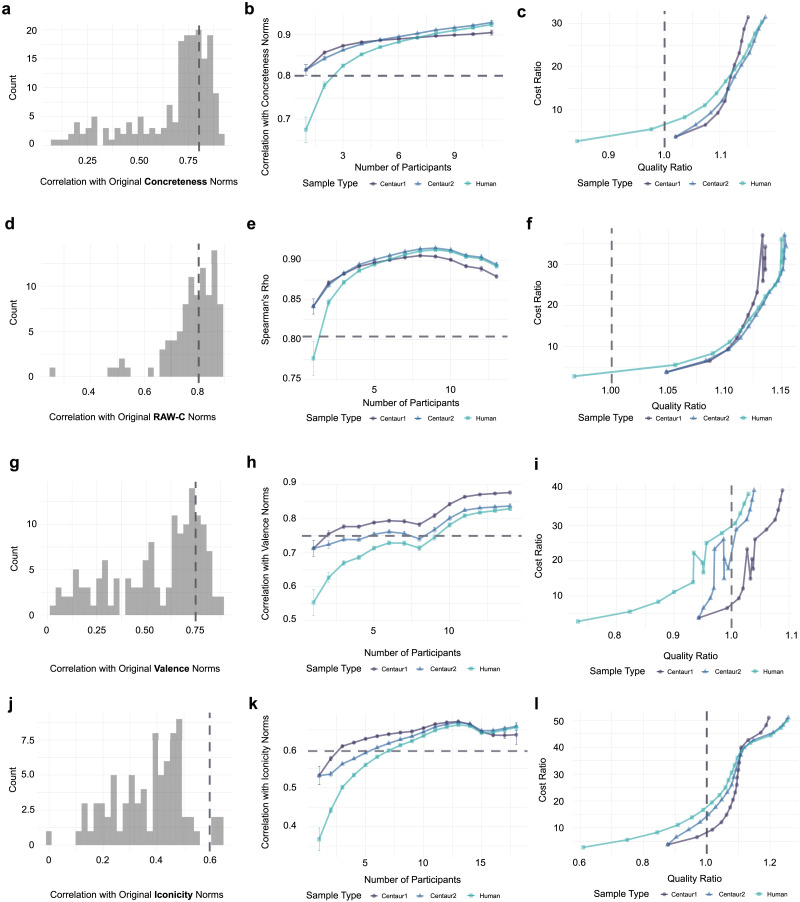
Top row represents the results for the Glasgow concreteness norms; second row represents the results for the RAW-C Norms; third row represents the results for the Glasgow valence norms; fourth row represents the results for the iconicity norms. (a), (d), (g), (j): distribution of individual participant correlations with the gold standard, as well as GPT-4’s correlation (dashed vertical blue line). (b), (e), (h), (k): correlation with gold standard as a function of sample size and sample type (e.g., which centaur method), compared to GPT-4’s correlation (dashed horizontal blue line). (c), (f), (i), (l): relationship between the cost ratio (human/centaur cost vs. LLM cost) and quality ratio (human/centaur quality vs. LLM quality); in each case, a value of 1 indicates parity between the experimental sample and cost (or quality) of the LLM.

We conducted three key analyses for each dataset. First, we computed the list-wise correlation for GPT-4, i.e., the extent to which GPT-4’s ratings correlated with the published ratings for that list of stimuli. We did the same for each participant for each list, which allowed us to compare GPT-4’s ratings to *individual* participant ratings (see Figures 1a, 1d, 1g, and 1j).

**Figure 2.**
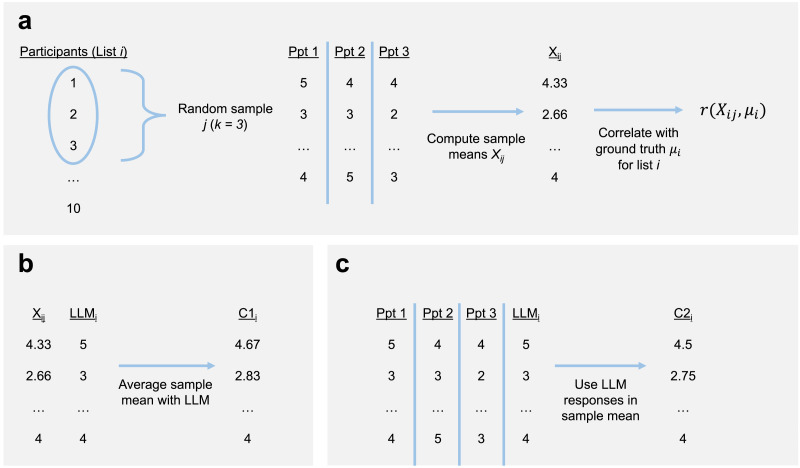
Graphical illustration of the procedure used for each sample type. (a) Standard human sample, in which judgments from a random subset of participants in list *i* are averaged and correlated with the gold standard. (b) Centaur 1 method, in which the mean of a given sample is averaged with the LLM’s judgments, which is then correlated with the gold standard. (c) Centaur 2 method, in which the LLM’s judgments are combined with the individual participant judgments as part of the sample mean; the average of these judgments is then correlated with the gold standard.

Second, we performed a sampling analysis (see Figure 2). Concretely, for each stimulus list, we sampled combinations of participants of sample size *k* (ranging from 1 to the total number of participants assigned to that list), calculated the sample mean, then measured the correlation between that sample mean and the gold standard. These measures were compared to GPT-4’s correlations with the gold standard to determine the Number Needed to Beat, or NNB. Also part of this analysis were the two “centaur” methods, in which GPT-4’s ratings were either averaged with the human sample’s mean (Centaur 1) or added as another “participant”, i.e., forming part of that sample mean calculation (Centaur 2).

Finally, we performed a cost/quality analysis. The quality ratios were obtained by dividing a given sample type’s correlation with the gold standard by GPT-4’s correlation with the gold standard. This was done for each sample size. The cost ratios were obtained by estimating the average cost per judgment for humans and for GPT-4, using assumptions about the hourly rate ($12 per hour), judgment duration (5 seconds per judgment), and the number of sampled and generated tokens for each GPT-4 judgment (20 and 10, respectively). Cost ratios were then calculated by comparing the number of human judgments needed for a given sample size by the human cost per judgment, and dividing that by GPT-4’s cost per judgment. The centaur cost ratios were identical except insofar as GPT-4’s cost per judgment was added to the numerator of the ratio.

As mentioned in the manuscript, the cost/quality analysis assumptions are included in the analysis code, allowing other researchers to try out various permutations; note that the judgment duration is likely a slight underestimate—for example, based on the completion times for the relatedness task, relatedness judgments took approximately 7.4 seconds each. Similarly, the hourly rate for humans ($12 per hour) is also an underestimate.

All analyses were performed in Python and R. Visualizations were produced using the *ggplot2* library (Wickham, 2006).

## RESULTS

### Dataset 1: Contextualized Concreteness

On a scale from 1 (most abstract) to 7 (most concrete), a sample of novel human participants and GPT-4 rated the concreteness of 379 ambiguous words in context (e.g., “bow (ship)” or “bow (arrow)”) for a total of 871 ratings overall. The stimuli were divided into 17 lists of approximately 50 words each; each human participant rated words from only a single list. These ratings were then compared with the “gold standard” ratings published in the Glasgow Norms (Scott et al., 2019).

Ratings elicited from GPT-4 exhibited high correlations with the original Glasgow concreteness Norms across each list tested (mean Spearman’s *rho* = 0.8, *SD* = 0.04)^6^. The degree of correlation between individual human judgments and the original norms was also significant, but comparatively lower (mean Spearman’s *rho* = 0.67, *SD* = 0.2). GPT-4’s degree of correlation with the gold standard was higher than approximately 70% of the human participants, even after applying our exclusion procedure (see also Figure 1a).

To determine the number of humans needed to match GPT-4’s correlation with the gold standard (i.e., the Number Needed to Beat, or NNB), we tested a range of sample sizes for each stimulus list. For each sample size, we took random combinations of participants to form a “sample” (e.g., if there are 10 participants to select from, the number of unique combinations of size 3 would be 120)^7^, computed their sample mean, and measured its correlation with the gold standard (see Figure 2a for a graphical illustration of this process). We then compared this to GPT-4’s correlation using both the difference and ratio. Finally, we asked at what sample size *k* this difference (or ratio) was significantly less than 0 (or 1), i.e., at which point the human sample mean’s correlation exceeded GPT-4’s. One-tailed t-tests indicated a NNB of three, regardless of whether the ratio [*t*(2688) = −9.46, *p* < .001] or difference [*t*(2688) = −18.81, *p* < .001] was used (see also Figure 1b).

Finally, we explored two “centaur” methods in which human data was *augmented* by GPT-4’s data (see Figure 2b and Figure 2c for a graphical illustration of these approaches). In the first method (Centaur 1), we computed the average of GPT-4’s rating and a given sample mean; in the second (Centaur 2), we added GPT-4’s ratings to a given sample and included those ratings in the calculation of its mean (effectively increasing the sample size by one). As depicted in Figure 1b, both centaur methods out-performed GPT-4 on its own; additionally, both methods out-performed human-only samples for samples smaller than six. As sample size increased beyond six, the first centaur method proved less effective, likely because GPT-4’s ratings were over-weighted.

### Dataset 2: Contextualized Relatedness Judgments

On a scale from 1 (totally different meanings) to 5 (same meaning), a sample of novel human participants and GPT-4 rated the relatedness of 112 ambiguous words across six sentence pairs each (e.g., “She liked the marinated *lamb*” vs. “She liked the friendly *lamb*”), for a total of 672 ratings overall. The stimuli were divided into 8 lists of 84 words each; each human participant rated words from only a single list. These ratings were then compared with the “gold standard” ratings published in RAW-C (Trott & Bergen, 2021).

GPT-4’s ratings were again highly correlated with the original norms (mean Spearman’s *rho* = 0.8, *SD* = 0.02), as were individual human judgments (mean Spearman’s *rho* = 0.78, *SD* = 0.1). We then performed the same analysis as for Dataset 1 to calculate the Number Needed to Beat GPT-4’s rating. One-tailed t-tests indicated a NNB of two, regardless of whether the ratio [*t*(516) = −17.21, *p* < .001] or difference [*t*(516) = −20.52, *p* < .001] was used (see also Figure 1e). As in the first analysis, we also explored the same two “centaur” methods. Again, both centaur methods out-performed GPT-4’s ratings alone, as well as human-only samples smaller than four. As sample size increased beyond four, the first centaur method offered diminishing returns and eventually impaired performance (again, likely because GPT-4’s norms were “over-weighted” in the resulting calculation).

**Figure A2.**
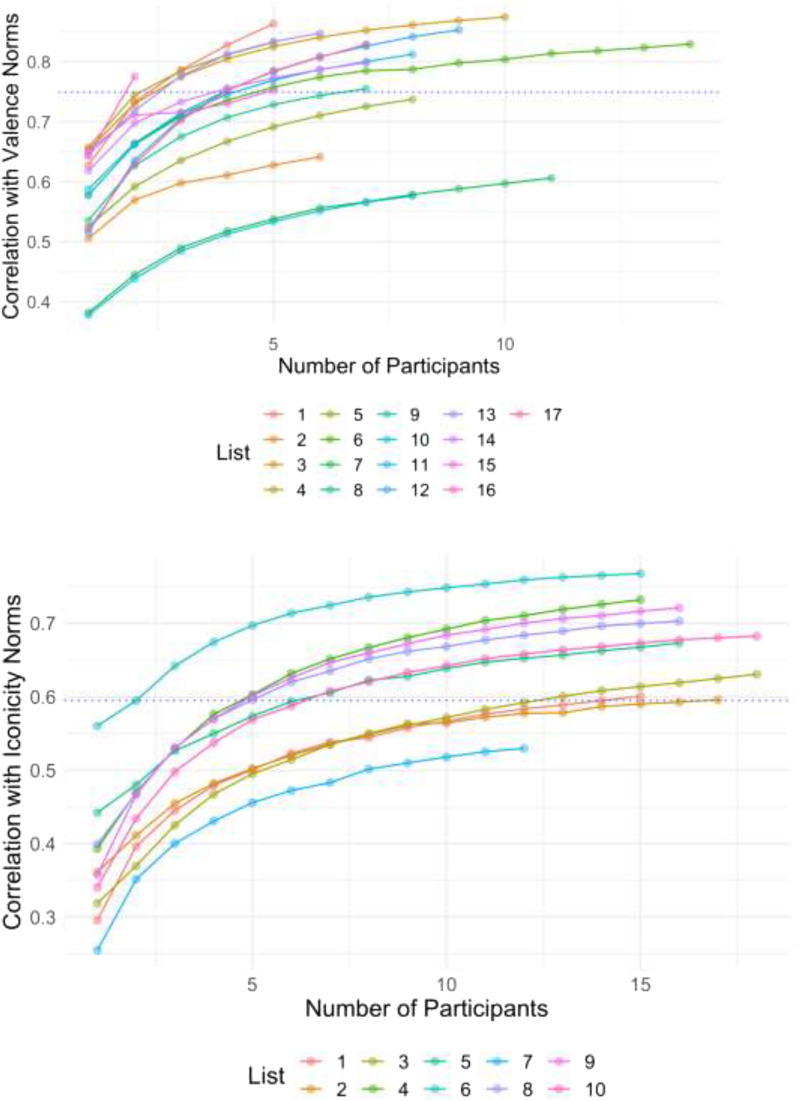
Correlation with gold standard valence (top) and iconicity (bottom) norms as a function of number of participants and list number. Within each list, larger samples showed the expected increase in correlation with the gold standard. However, there was some across-list variation in the average correlation.

Note that the apparent lack of monotonicity between human sample size and correlation with the gold standard (both here, and for the contextualized valence and iconicity datasets) is driven by list-wise variation in correlations with the gold standard and unbalanced random assignments to each list; see Appendix (and Figure A2) for a more thorough description.

### Dataset 3: Contextualized Valence Judgments

On a scale from 1 (very negative) to 7 (very positive), a sample of novel human participants and GPT-4 rated the valence of 379 ambiguous words in context (e.g., “mean (nasty)” or “mean (average)”) for a total of 871 ratings overall. As with the Glasgow concreteness norms, the stimuli were divided into 17 lists of approximately 50 words each; each human participant rated words from only a single list.

GPT-4’s ratings were highly correlated with the ratings from the originally published Glasgow Norms across each list tested (mean *rho* = 0.75, *SD* = 0.07). This was higher than the average correlation between individual human judgments and the original norms (mean *rho* = 0.55, *SD* = 0.23). GPT-4’s performance was higher than approximately 79.6% of the human participants, even after applying our exclusion procedure (see also Figure 1g).

We then performed the same analysis as for Datasets 1–2 to calculate the Number Needed to Beat GPT-4’s rating. Using Spearman’s *rho*, the NNB was 11, regardless of whether the ratio [*t*(511) = −2.17, *p* = 0.01] or difference [*t*(511) = −3.68, *p* < .001] was used; using Pearson’s *r*, there was no sample size whose performance exceeded GPT-4’s. Both centaur methods consistently out-performed human samples. The first centaur method out-performed GPT-4 for sample sizes larger than 2, and the second centaur method out-performed GPT-4 consistently only for sample sizes larger than approximately six (see Figure 1h).

### Dataset 4: Iconicity Judgments

On a scale from 1 (not at all iconic) to 7 (very iconic), a sample of human participants and GPT-4 rated the iconicity of 800 English words in isolation. Each participant was randomly assigned to one of 10 lists of 80 words and was also given the option to indicate that they did not know a given word, as in the original study (Winter et al., 2024).

As previously reported (Trott, 2024), GPT-4’s ratings exhibited relatively high correlation with the gold standard ratings (Winter et al., 2024) across each list tested (mean *rho* = 0.595, *SD* = 0.1). Although this was lower than the correlation for other datasets, it was still higher than the average correlation between individual human judgments and the gold standard (mean *rho* = 0.37, *SD* = 0.13); see also Figure 1j for a visual comparison of the distribution of individual human correlations and GPT-4’s correlation.

We then calculated NNB using the same analyses as in previous datasets. Here, the precise number varied depending on exactly which measure of correlation was used and whether the statistical test was done on *differences* or *ratios* between GPT-4’s and human judgments. Using Spearman’s *rho*, the NNB was 5 for the ratio [*t*(4995) = −2.82, *p* = 0.002] and 8 for the difference [*t*(4999) = −14.3, *p* < .001]; using Pearson’s *r*, the NNB was 7 for both the ratio [*t*(4999) = −1.8, *p* = .03] and the difference [*t*(4999) = −5.02, *p* < .001].

Finally, both centaur methods consistently out-performed human samples up to samples of approximately 10, though the first centaur method was more effective for smaller samples. For larger samples (e.g., *N* = 15), the first centaur method was less effective than either human samples alone or the second centaur method.

### Cost/Quality Trade-Off Analysis

One argument for replacing (or augmenting) human participants with LLMs is cost-effectiveness. But while LLMs might be cheaper, if their quality is compromised, they might not offer value for researchers. Decisions about whether to use an LLM should be informed by both quality (e.g., the NNB) and cost differences. We evaluated the cost-quality trade-off with increasing human sample size. For each sample size, we calculated the cost and quality ratio relative to an LLM, where values below 1 suggest cost-effectiveness or lower quality and above 1 indicate the opposite. Human judgments were priced at a rate of $12/hour, assuming 5-second judgments^8^ (i.e., about $0.017 per judgment); GPT-4’s judgments were priced at a rate of $0.0003 per 1000 sampled tokens and $0.0006 per 1000 generated tokens, assuming approximately 20 sampled and 10 generated tokens per judgment.

The relationship between these variables is depicted for each dataset in Figure 1c (for contextualized concreteness), Figure 1e (for contextualized relatedness), Figure 1h (for contextualized valence), and Figure 1k (for iconicity). Each figure reveals the same core insight: higher-quality data can eventually be achieved with human samples, though at considerably higher cost. In fact, for the Glasgow concreteness norms, one must pay almost 10x as much to beat the data quality provided by GPT-4. For the Glasgow valence norms, one must pay over 30x as much to beat GPT-4’s performance.

Each figure also reflects the unique advantages of the centaur methods. Figures 1c and 1f demonstrate when and why the second centaur method (Centaur 2) is useful: unlike Centaur 1, Centaur 2’s data quality continues to improve with sample size (like the human sample), but at least for smaller samples, costs less to achieve comparable data quality. Thus, Centaur 2 is a good option when NNB is relatively small, because it does not “over-weight” GPT-4’s ratings. In contrast, Centaur 1 out-performs Centaur 2 in Figure 1i. Thus, Centaur 1 may be a particularly good option when NNB is larger—because GPT-4’s ratings continue to be of higher quality than human samples until NNB is reached, better performance is achieved by assigning a larger weight to GPT-4’s ratings than individual human ratings. At the same time, the relative benefit of “over-weighting” GPT-4’s ratings generally diminishes (and reverses) once the human sample is of sufficiently large size, as depicted in Figures 1c, 1f, and 1l.

## GENERAL DISCUSSION

We developed and presented a novel methodological framework for assessing the viability of augmenting human datasets with LLMs. This framework estimates the number of human participants needed to approximate the quality of the sample statistic provided by GPT-4, a state-of-the-art LLM. The “number needed to beat” (NNB) GPT-4 can be used to inform decisions about whether to incorporate LLMs into the data collection process. We applied this framework to four datasets, which revealed different NNB values for different tasks (ranging from 2 to 11). We also introduced novel “centaur” methods, which combined human-generated and LLM-generated data, and found that these centaur methods were effective ways to both improve data quality and reduce costs (see Figure 1c, Figure 1f, Figure 1i, and Figure 1l). Further, different centaur methods may be appropriate depending on whether NNB is high or low.

This work informs broader debates about whether LLMs can accelerate scientific progress (Korinek, 2023), specifically by replacing samples of human participants (Dillion et al., 2023; Grossmann et al., 2023). Some have proposed that LLMs capture the combined intelligence of many individual humans (Dillion et al., 2023), while others have argued that LLMs cannot and should not replace humans as subjects (Harding et al., 2023). The current work contributes to this debate by providing an empirical framework for evaluating whether and to what extent an LLM captures the “wisdom of the crowd”.

As we report, NNB varies considerably across types of tasks: in some cases (e.g., contextualized relatedness), NNB is fairly small (2), and in others (e.g., contextualized valence), it was larger (> 10). In terms of the original theoretical question we set out to address—namely, whether LLMs capture the “wisdom of the crowd”—these results point to three tentative conclusions. First, at least on these datasets, LLMs do reflect the aggregate “wisdom” of more than one human annotator; second, the “crowd” captured in these judgments is not particularly large (ranging from 2–11); and third, the size of the crowd depends on the kind of task, perhaps reflecting variance in how easily a kind of task can be learned from the statistics of language use.

In terms of practical applications, a crucial question concerns the reliability and replicability of NNB. Clearly, NNB varies *across* tasks—even within a language—suggesting that it would be unwise to rely on the NNB obtained for one task (e.g., iconicity judgments) when making decisions about a different kind of task (e.g., contextualized relatedness judgments). A key concern moving forward will be to establish the reliability of NNB *within* a given task. If NNB is fairly consistent for a certain kind of task (e.g., contextualized relatedness) across different stimuli within a given language, this information could be used by researchers for future applications of that task; however, if NNB depends too much on characteristics of the specific implementation or even the specific words that were used, then this method may have limited applicability when collecting novel data (see Appendix). Individual researchers could estimate NNB using a small sample of human participants on a subset of their data, which may inform their decisions about whether and to what extent to augment their data with an LLM. It is also possible that some tasks are more similar than others; future work could aim to more thoroughly taxonomize this variation, with the hope of producing a generalizable theory of which *types* of tasks one would expect a low vs. high NNB. Notably, NNB matters not just for determining the size of one’s sample, but also about which type of “centaur” method may be most useful: where NNB is small, the second centaur method is more useful (i.e., treating the LLM as one participant of many); where NNB is large, the first centaur method may be more cost-effective (i.e., over-weighting the LLM judgments). Finally, these analyses were all conducted on English words or word phrases using an LLM trained primarily on written English; it is unclear—and perhaps unlikely—that the NNB calculated for a task in English would generalize to the same task in other languages.

Another key concern relates to the *representativeness* of both human samples and LLM-generated data. The majority of psychology research draws from a biased set of “WEIRD” (Western, Educated, Industrialized, Rich, Democratic) human participants (Henrich et al., 2010); recent work suggests that contemporary LLMs like GPT-4 are “WEIRD” as well (Atari et al., 2023), presumably because they are trained on data produced by an unrepresentative sample of language users (Bender et al., 2021; Crockett & Messeri, 2023). There is some evidence that this can be partially ameliorated using prompting methods or fine-tuning (Ramezani & Xu, 2023), though future work could aim to quantify the limits of this approach. Crucially, one lesson of this research (Henrich et al., 2010) is not just that a sample statistic should include a broader pool of participants, but rather that in many cases, the “average” is not a coherent statistic to measure when there are distinct populations of interest. Beyond the question of “how many humans” an LLMs represents, then, an equally important question is “*which* humans” (Atari et al., 2023).

Indeed, LLMs may be poorly suited to questions about individual *variation* (Harding et al., 2023), for precisely the same reasons that others have argued they capture the “wisdom of the crowd” (Dillion et al., 2023): the central tendency of a sample collapses across the variation in that sample (Gould, 2013). Some research has explored whether LLMs can be adapted to this purpose (Argyle et al., 2023), though the degree of success may depend on a researcher’s desired degree of granularity. Individual variation is at the heart of many scientific questions, and this fact should not be ignored in the search for a more precise estimate of a sample’s central tendency. It is an open question whether different prompting techniques, or different temperature settings, can induce stochasticity in LLM responses that resembles variance in human judgments—or whether LLMs are simply ill-suited to questions of individual differences.

Ultimately, decisions about whether and to what extent to use LLMs as sources of behavioral data will be informed by a field’s values and research questions, as well as relevant empirical data. The current research is empirical in nature, offering a method for establishing the “size” of an LLM’s alleged sample mean (NNB) and the extent to which this sample mean is a biased estimate of the population parameter. Crucially, interested researchers can and should apply this analytical framework to their specific research question: LLMs may be a viable augmentation strategy for some tasks and not others, and this framework is a step towards navigating that decision.

## ACKNOWLEDGMENTS

Thank you to Benjamin Bergen, Cameron Jones, and Pamela Rivière for valuable discussions about this work. Thank you to Pamela Rivière specifically for help with the figures.

## Notes

^1^ In at least one case, this has motivated the creation of a company (https://www.syntheticusers.com/).^2^ Of course, recent work (Henrich et al., 2010) has demonstrated that in practice, samples are often *not* randomly drawn from the underlying population. Nevertheless, the underlying statistical premise is still sound.^3^ In other ways, an LLM’s input is much smaller, e.g., most LLMs do not have access to embodied, physical experience or social interaction (Bender & Koller, 2020).^4^ There are some reports that for some tasks, behavior is not fully deterministic even at a temperature of 0; this might relate to the use of sparse mixture of experts in the token sampling process (https://152334h.github.io/blog/non-determinism-in-gpt-4/).^5^ Note that we intended to recruit 100 participants for RAW-C, but due to a sampling mismatch between Gorilla and Prolific, we ended up sampling 102.^6^ Note that all results are qualitatively identical for the concreteness Norms if Pearson’s *r* is used instead of Spearman’s *rho*. For RAW-C, GPT-4’s Pearson’s correlation with the gold standard is statistically indistinguishable from the average individual’s Pearson’s correlation (i.e., NNB = 1). Additionally, for the valence norms and the iconicity norms, NNB varied according to which score metric was used. The appropriateness of a given correlation measure will depend on the structure of the data (e.g., whether there are outliers) and the relationship being tested (e.g., similar rank orderings vs. linear relationships). Where there are disparities, we have reported both, so that researchers can develop their own “rules of thumb”: for example, it might make sense to use the score metric that results in a lower NNB, so that one does not overestimate the relative quality of an LLM’s judgments.^7^ If there were more than 500 samples of size *N* for a given list, we selected a random subset of 500 participant combinations.^8^ Note that it is possible 5 seconds per judgment is a slight underestimate; see Methods for more details. The code for estimating the cost ratios is included in the publicly available GitHub, so readers can plug in their own values.

## APPENDIX: ANALYSIS 1: LIST-WISE VARIATION

One question that arose in the primary manuscript was why for certain datasets (e.g., contextualized valence), the performance of human samples exhibited apparently non-monotonic relationships with sample size—a surprising result, given that larger sample sizes should on average yield better, more precise estimates of the population parameter. Closer inspection revealed that this was in fact due to two factors: 1) different *lists* of items (to which participants were randomly assigned) had, on average, different degrees of correlation with the gold standard; and 2) although participants were randomly assigned to lists, this process was not perfectly *balanced*, such that some lists had more participants on average. This meant that some lists had as many as fourteen observations per item, while others had as few as six or seven. Therefore, the *average* performance across all lists might appear non-monotonic if some of the best-performing lists had fewer items than other, worse-performing lists.

A clear illustration of this effect can be seen by focusing solely on the human samples, then plotting the correlation between a given list at a given sample size with the gold standard. Below, this is illustrated for the contextualized valence norms and the iconicity norms. In both cases, there is a monotonic relationship *within* each list between sample size and correlation with the gold standard. However, some lists perform noticeably better than others.

I then conducted a follow-up analysis for the contextualized valence norms to ask what, if anything, predicted this list-wise variation. Interestingly, list-wise variance in GPT-4’s correlation with the gold standard was positively correlated with list-wise variance in each human sample’s correlation with the gold standard (*r* = 0.54, *p* = 0.02). However, a linear model including other features of the lists themselves (e.g., mean valence, standard deviation of valence *across* words, and mean standard deviation of valence *within* each word) did not yield any significant predictors.

## References

[bib1] Aher, G., Arriaga, R. I., & Kalai, A. T. (2023). Using large language models to simulate multiple humans and replicate human subject studies. In Proceedings of the 40th international conference on machine learning (pp. 337–371). PMLR.

[bib2] Anwyl-Irvine, A. L., Massonnié, J., Flitton, A., Kirkham, N., & Evershed, J. K. (2020). Gorilla in our midst: An online behavioral experiment builder. Behavior Research Methods, 52(1), 388–407. 10.3758/s13428-019-01237-x, 31016684 PMC7005094

[bib3] Argyle, L. P., Busby, E. C., Fulda, N., Gubler, J. R., Rytting, C., & Wingate, D. (2023). Out of one, many: Using language models to simulate human samples. Political Analysis, 31(3), 337–351. 10.1017/pan.2023.2

[bib4] Atari, M., Xue, M. J., Park, P. S., Blasi, D., & Henrich, J. (2023). Which humans? PsyArXiv. 10.31234/osf.io/5b26t

[bib7] Bender, E. M., Gebru, T., McMillan-Major, A., & Shmitchell, S. (2021). On the dangers of stochastic parrots: Can language models be too big? In Proceedings of the 2021 ACM conference on fairness, accountability, and transparency (pp. 610–623). Association for Computing Machinery. 10.1145/3442188.3445922

[bib34] Bender, E. M., & Koller, A. (2020). Climbing towards NLU: On meaning, form, and understanding in the age of data. In Proceedings of the 58th annual meeting of the Association for Computational Linguistics (pp. 5185–5198). Association for Computational Linguistics. 10.18653/v1/2020.acl-main.463

[bib5] Brown, T. B., Mann, B., Ryder, N., Subbiah, M., Kaplan, J., Dhariwal, P., Neelakantan, A., Shyam, P., Sastry, G., Askell, A., Agarwal, S., Herbert-Voss, A., Krueger, G., Henighan, T., Child, R., Ramesh, A., Ziegler, D. M., Wu, J., Winter, C., … Amodei, D. (2020). Language models are few-shot learners. In Proceedings of the 34th international conference on neural information processing systems (pp. 1877–1901). Curran Associates Inc.

[bib6] Brysbaert, M., Stevens, M., De Deyne, S., Voorspoels, W., & Storms, G. (2014). Norms of age of acquisition and concreteness for 30,000 Dutch words. Acta Psychologica, 150, 80–84. 10.1016/j.actpsy.2014.04.010, 24831463

[bib8] Crockett, M. J., & Messeri, L. (2023). Should large language models replace human participants? PsyArXiv. 10.31234/osf.io/4zdx9

[bib10] Dietterich, T. G. (2000). Ensemble methods in machine learning. In J. Kittler & F. Roli (Eds.), Multiple classifier systems: First international workshop, MCS 2000, Cagliari, Italy, June 2000, Proceedings (pp. 1–15). Springer. 10.1007/3-540-45014-9_1

[bib35] Dillion, D., Tandon, N., Gu, Y., & Gray, K. (2023). Can AI language models replace human participants? Trends in Cognitive Sciences, 27(7), 597–600. 10.1016/j.tics.2023.04.008, 37173156

[bib11] Gilardi, F., Alizadeh, M., & Kubli, M. (2023). ChatGPT outperforms crowd-workers for text-annotation tasks. Proceedings of the National Academy of Sciences, 120(30), e2305016120. 10.1073/pnas.2305016120, 37463210 PMC10372638

[bib12] Gould, S. J. (2013). The median isn’t the message. Virtual Mentor, 15(1), 77–81. 10.1001/virtualmentor.2013.15.1.mnar1-1301, 23356812

[bib13] Grossmann, I., Feinberg, M., Parker, D. C., Christakis, N. A., Tetlock, P. E., & Cunningham, W. A. (2023). AI and the transformation of social science research. Science, 380(6650), 1108–1109. 10.1126/science.adi1778, 37319216

[bib14] Hagendorff, T., Fabi, S., & Kosinski, M. (2023). Human-like intuitive behavior and reasoning biases emerged in large language models but disappeared in ChatGPT. Nature Computational Science, 3(10), 833–838. 10.1038/s43588-023-00527-x, 38177754 PMC10766525

[bib15] Harding, J., D’Alessandro, W., Laskowski, N. G., & Long, R. (2023). AI language models cannot replace human research participants. AI & Society. 10.1007/s00146-023-01725-x

[bib16] Hart, B., & Risley, T. R. (1992). American parenting of language-learning children: Persisting differences in family-child interactions observed in natural home environments. Developmental Psychology, 28(6), 1096–1105. 10.1037/0012-1649.28.6.1096

[bib17] Henrich, J., Heine, S. J., & Norenzayan, A. (2010). The weirdest people in the world? Behavioral and Brain Sciences, 33(2–3), 61–83. 10.1017/S0140525X0999152X, 20550733

[bib18] Hosseini, E. A., Schrimpf, M., Zhang, Y., Bowman, S., Zaslavsky, N., & Fedorenko, E. (2024). Artificial neural network language models predict human brain responses to language even after a developmentally realistic amount of training. Neurobiology of Language, 5(1), 43–63. 10.1162/nol_a_00137, 38645622 PMC11025646

[bib19] Jain, S., Vo, V. A., Wehbe, L., & Huth, A. G. (2024). Computational language modeling and the promise of in silico experimentation. Neurobiology of Language, 5(1), 80–106. 10.1162/nol_a_00101, 38645624 PMC11025654

[bib20] Korinek, A. (2023). Language models and cognitive automation for economic research (Working Paper No. 30957). National Bureau of Economic Research. 10.3386/w30957

[bib21] Lynott, D., Connell, L., Brysbaert, M., Brand, J., & Carney, J. (2020). The Lancaster Sensorimotor Norms: Multidimensional measures of perceptual and action strength for 40,000 English words. Behavior Research Methods, 52(3), 1271–1291. 10.3758/s13428-019-01316-z, 31832879 PMC7280349

[bib22] Palan, S., & Schitter, C. (2018). Prolific.ac—A subject pool for online experiments. Journal of Behavioral and Experimental Finance, 17, 22–27. 10.1016/j.jbef.2017.12.004

[bib23] Ramezani, A., & Xu, Y. (2023). Knowledge of cultural moral norms in large language models. arXiv. 10.48550/arXiv.2306.01857

[bib24] Scott, G. G., Keitel, A., Becirspahic, M., Yao, B., & Sereno, S. C. (2019). The Glasgow Norms: Ratings of 5,500 words on nine scales. Behavior Research Methods, 51(3), 1258–1270. 10.3758/s13428-018-1099-3, 30206797 PMC6538586

[bib25] Sourati, J., & Evans, J. A. (2023). Accelerating science with human-aware artificial intelligence. Nature Human Behaviour, 7(10), 1682–1696. 10.1038/s41562-023-01648-z, 37443269

[bib26] Stroop, J. R. (1932). Is the judgment of the group better than that of the average member of the group? Journal of Experimental Psychology, 15(5), 550–562. 10.1037/h0070482

[bib27] Trott, S. (2024). Can large language models help augment English psycholinguistic datasets? Behavior Research Methods. 10.3758/s13428-024-02337-z, 38261264 PMC11335796

[bib28] Trott, S., & Bergen, B. (2021). RAW-C: Relatedness of Ambiguous Words in Context (A new lexical resource for English). In C. Zong, F. Xia, W. Li, & R. Navigli (Eds.), Proceedings of the 59th annual meeting of the Association for Computational Linguistics and the 11th international joint conference on natural language processing (Volume 1: Long papers) (pp. 7077–7087). Association for Computational Linguistics. 10.18653/v1/2021.acl-long.550

[bib29] Trott, S., Jones, C., Chang, T., Michaelov, J., & Bergen, B. (2023). Do large language models know what humans know? Cognitive Science, 47(7), e13309. 10.1111/cogs.13309, 37401923

[bib30] Veselovsky, V., Ribeiro, M. H., & West, R. (2023). Artificial artificial artificial intelligence: Crowd workers widely use large language models for text production tasks. arXiv. 10.48550/arXiv.2306.07899

[bib32] Wickham, H. (2006). An introduction to ggplot: An implementation of the grammar of graphics in R. Springer.

[bib33] Winter, B., Lupyan, G., Perry, L. K., Dingemanse, M., & Perlman, M. (2024). Iconicity ratings for 14,000+ English words. Behavior Research Methods, 56(3), 1640–1655. 10.3758/s13428-023-02112-6, 37081237

